# Noninvasive Cardiac Flow Assessment Using High Speed Magnetic Resonance Fluid Motion Tracking

**DOI:** 10.1371/journal.pone.0005688

**Published:** 2009-05-25

**Authors:** Kelvin Kian Loong Wong, Richard Malcolm Kelso, Stephen Grant Worthley, Prashanthan Sanders, Jagannath Mazumdar, Derek Abbott

**Affiliations:** 1 Centre for Biomedical Engineering and School of Electrical & Electronic Engineering, University of Adelaide, Adelaide, South Australia; 2 School of Mechanical Engineering, University of Adelaide, Adelaide, South Australia; 3 School of Medicine, University of Adelaide, Adelaide, South Australia; Cornell University, United States of America

## Abstract

Cardiovascular diseases can be diagnosed by assessing abnormal flow behavior in the heart. We introduce, for the first time, a magnetic resonance imaging-based diagnostic that produces sectional flow maps of cardiac chambers, and presents cardiac analysis based on the flow information. Using steady-state free precession magnetic resonance images of blood, we demonstrate intensity contrast between asynchronous and synchronous proton spins. Turbulent blood flow in cardiac chambers contains asynchronous blood proton spins whose concentration affects the signal intensities that are registered onto the magnetic resonance images. Application of intensity flow tracking based on their non-uniform signal concentrations provides a flow field map of the blood motion. We verify this theory in a patient with an atrial septal defect whose chamber blood flow vortices vary in speed of rotation before and after septal occlusion. Based on the measurement of cardiac flow vorticity in our implementation, we establish a relationship between atrial vorticity and septal defect. The developed system has the potential to be used as a prognostic and investigative tool for assessment of cardiac abnormalities, and can be exploited in parallel to examining myocardial defects using steady-state free precession magnetic resonance images of the heart.

## Introduction

Congenital cardiovascular defects can contribute to heart failures, and various screening methods for cardiovascular diseases exist in the medical community. Some of the common imaging modalities for detecting heart defects are electrocardiography, echocardiography, and chest radiography. In this paper, we examine the use of cardiac magnetic resonance imaging (MRI) to examine such heart abnormalities.

Cardiac examination and management of patients remain the key priorities in treatment of heart defects. However, the need for a concise insight into a given defect is desired for diagnosis. For example, cardiac examination can be based on assessment of myocardial viability from magnetic resonance images [1], [2], [3] or electrocardiograms for assessing abnormal electrical activity within the heart in the treatment of cardiac arrhythmia [4], [5]. A good cardiac diagnostic system will have the ability to provide information about the heart that can be utilized for effective analysis depending on the nature of the diagnosis. The high rate of mortality due to cardiovascular diseases in developed countries gives rise to a growing requirement in the market for diagnostic tools and therapeutic devices for the treatment of cardiovascular diseases. Appropriate and timely intervention by cardiologists can reduce mortality from heart diseases. Selection of medical treatment and methods of intervention, particularly in borderline cases, is important and appropriate diagnostic tools will augment medical expertise in terms of disease or cardiovascular defect classification.

We propose that it will be of clinical interest to apply flow analysis within heart in order to examine cardiovascular heart diseases. It is widely appreciated that flow characteristics in the left atrium is important in activities of the heart. Blood shearing in turbulent flow induces platelet activation and contributes to thrombus formation [6]–[8]. In other cases, blood clots are formed in the setting of blood stagnation and pooling within a non-contractile atrium of a heart with a condition such as atrial fibrillation. Consequently, a high incidence of left atrial thrombus results in higher risk of stroke in such patients [9]. Therefore, irregular blood flow can lead to blood clots and stroke, so cardiac patients may require lifelong therapies. This motivates the need for cardiac flow measurement and visualization systems in practice. Flow visualization within the human heart augments the percipience and experience of cardiologists and can assist in the understanding of the genesis and progression of cardiac abnormalities. Quantitative prediction or diagnosis of cardiac failures is of importance for clinical application. Medical organizations will be able to assess the degree of cardiac abnormality in the heart and arteries so that appropriate medical action can be taken.

Current methodologies, based on nuclear signals and pertaining to phase sensitive flow, enable velocity fields to be encoded within cine MR images, which are then post-processed during cardiac flow field mapping [10], [11]. The concept of contrasting the phases of imaged nuclei spins, during MRI measurement intervals, gives this system its commonly accepted terminology - phase contrast magnetic resonance imaging (PCMRI). It is essentially a technology that enables flow visualization in cardiac structures by means of subtraction of flow-sensitized data from reference volumes during the processing stage [12]. However, because of inherent limitations, such as undesirable measurement duration and sub-optimal temporal resolution, time-resolved three dimensional data acquisition has been mainly limited to the study of cardiac operations in research environments and not so much for diagnostic applications. Other complications exist at the present time, for example, respiration artifacts should be minimized as much as possible, while achieving a tolerable total scan time for patients [13].

Although phase contrast MRI scanning can perform flow visualization in the human heart, it has not been designed for analysis and characterization of blood flow in cardiac structures. Therefore, there is a need for a dedicated protocol in the shift of processing time and resources during MR imaging to the post-processing stage for the assessment of cardiac blood flow effectively without compromising the accuracy of flow information retrieval to an unrealistic extent. Such technology development can facilitate the prognosis or diagnosis of cardiac abnormalities.

Blood flow visualization can reveal vital information in the cardiac chambers and arteries. Interesting phenomena such as vortices in the heart's ventricular chambers and aorta have been studied previously [14]–[16], and how they affect heart valve operation [17] can thereby be examined and quantified. The quantification of flow characteristics within the heart and arteries provides vital information to cardiologists, who are interested in a range of problems from the hemodynamics of blood to biological phenomena in the heart. The scales and coherence of the vortex structures can be examined, and their existence can ultimately be linked to the operation of a number of cardiac structures influencing flow in the heart.

Cardiac flow analysis systems have the potential to be used as diagnostic devices for the assessment of heart disease and practical tools for physiological analysis [18]. There are potential applications for identifying risks after heart valve implant as well as determining the degree of atrial and ventricular septal defects. Septal defects (SD) may be detected in the adult by physical examination, electrocardiogram, chest radiograph, echocardiogram, or MRI [19]. Functional medical imaging is able to provide an alternative method for measurement and quantification of the blood flow rotation (defined as vorticity) in the heart of a patient diagnosed with SD. Rate of blood rotation is related to pressure within the heart chamber, and can be used to assess the degree of septal defect before and after septal occluder insertion. Such a method is particularly useful for evaluating the risk inflicted on the patients diagnosed with such cardiovascular diseases before and after surgical operation, and therefore has high clinical benefit for medical doctors parallel to examining the physical defect of the septum. Our approach is to measure flow and quantify its characteristics using typical steady-state free precession magnetic resonance images, making it attractive for future clinical practice.

## Results

Medical examiners and/or radiologists usually confirm the existence of septal defects by observation of a left to right shunt of blood through the septum using cine magnetic resonance images and also from the broken definition of the cardiac wall between two chambers [20]. Both indications of abnormality can be detected based on the intensity of the image signals captured on steady state free precession (SSFP) MRI scans. A variant of SSFP is true fast imaging in steady state free precession (True FISP) MRI [21], which is a protocol capable of imaging cross-sections of cardiac structures with unsurpassed soft tissue contrast. It is one of the most popular medical imaging modalities for the registration of physiological properties of the heart and arteries. This MRI protocol combines both longitudinal and transverse magnetization. It is characterized by complex T_1_/T_2_-contrast configurations and refocuses all gradients over a repetition interval, thereby achieving fast-imaging with a high signal. Magnetic resonance imaging based on the SSFP protocol has been suggested for the scans.

### Investigation of Sample Case Study Subject

**Figure 1:**
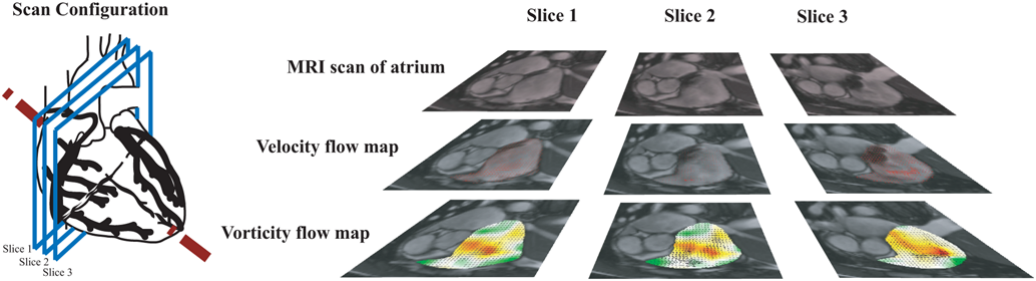
Imaging of heart based on short axis scan configuration. Steady-state free precession magnetic resonance image scans of the cardiac chambers were performed based on the short axis view at slice intervals of 6 mm, and labeled as Slices 1, 2 and 3 starting from the top to apex of the heart. Velocity and vorticity maps can be determined from the MR motion flow field for every slice and time frame.

We propose using magnetic resonance fluid motion tracking for examination of angular velocity in the right atrial flow. The tracking of blood flow within the chamber can be used to assess the severity of the cardiac abnormality in patients with atrial septal defects. MR image slices from the contiguous scans, in short axis views, through the ASD are presented (Fig. 1). Analyzes of atrial flow are carried out during the diastolic phases of the heart. The in-plane resolution of the scans is determined by the pixel spacing (at 1.33 mm/pixel and 1.67 mm/pixel for pre- and post-ASO images) and its through-plane resolution is based on a slice interval of 6 mm (for both images). The planes were chosen to be approximately normal to the mean flow direction.

A review of the right atrium from these scans has been performed. The selected MR images that correspond to the sectioning of the atria are labeled as slices numbering from 1 to 3, starting from top of the heart down to its apex through an axis oblique to the direction from head to foot, for examination and flow analyzes. The study for examination of vorticity in post-ASO condition applies the same order of sequence for the patient as during the pre-ASO scan. Vorticity maps of the segmented atrium based on a selection of 4 out of 25 phases of cardiac cycle for time frames *n_t_* = 10 to 13 are presented. From preliminary observations, we can deduce that there is a difference in strength of the vortical flow (Supplementary Videos 1 and 2), which is highlighted in more detail in the subsequent results.

**Figure 2:**
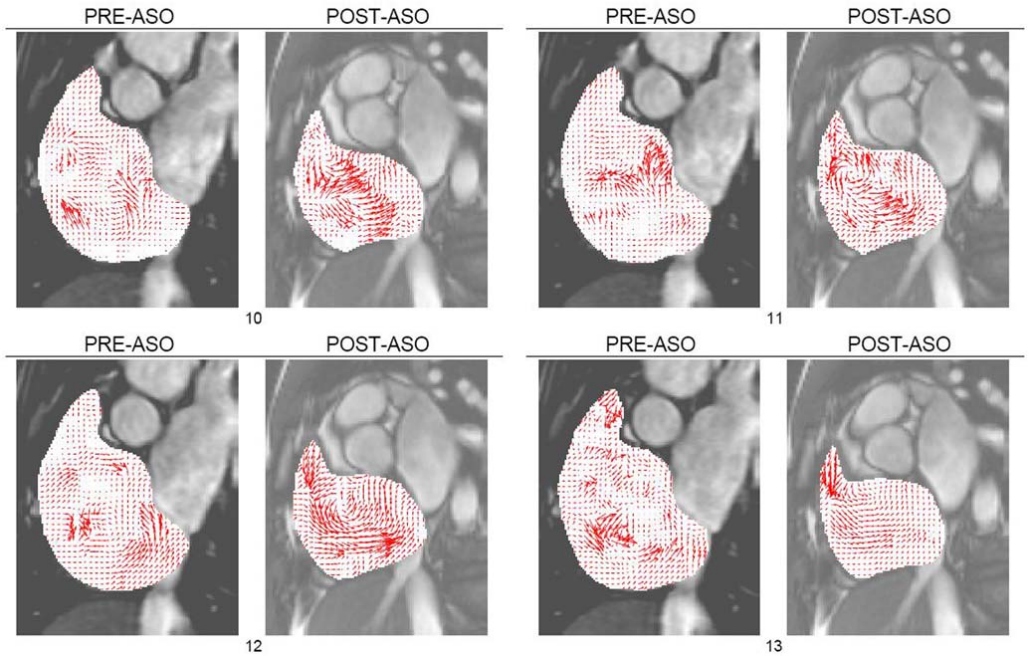
MR fluid motion flow fields of right atrium. The motion field of blood can be produced rapidly by using MR fluid motion tracking to give an estimation of the direction and magnitude of flow. The method can give a quick insight into the fluid flow behavior, and the swirling of blood based on cardiac phases at frames *n_t_* = [10, 11, 12, 13] can be observed more clearly after atrial septal occlusion. The swirling traced within the chamber region of interest shows the existence of vortices.

**Figure 3:**
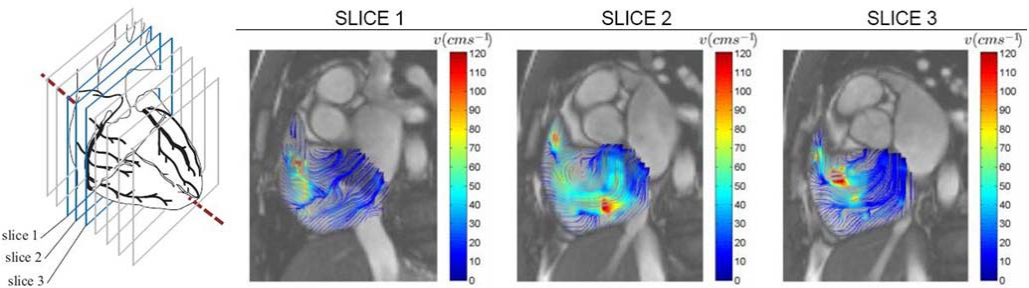
Slices 1 to 3 based on time frame 12 of post-atrial septal occlusion (ASO) scans. Color streamlines demonstrating blood swirl within segmented right atrium for post-ASO at three scan slices of heart is displayed here. Their vortices are presented for three consecutive slices. The reconstruction of these planar information can give a three dimensional perspective of the helix in the right atrium.

Vector field mapping of the flow in the sectional planes is presented for pre- and post-ASO (Fig. 2) based on the selected cardiac phases. Streamline tracing is performed for post-ASO flow fields (Fig. 3). This visual tool allows perception of the location and strength of the vortices in the right atrium. We highlight the poor tracking of blood made by the implemented technique, which is mainly due to the poor quality of tracking features present in the image. This demonstrates a limitation of MR fluid motion tracking as compared with phase contrast MR velocimetry. Nevertheless, the motion tracking technique makes it possible to show both quickly and conveniently that a more consistent vortical structure is present for the post-ASO case.

From Figure 2, we can visually deduce that the vortical features in the post-ASO atrium are more concentrated and coherent than those in the pre-ASO. Furthermore, the size and magnitude of the vorticity concentrations given in vorticity maps indicate the strength of blood rotation during one cardiac cycle [15]. Comparison of the pre- and post-surgical intervention based on the right atrial flow patterns and vorticity maps can provide an indication of the cardiac abnormality as well as the recovery conditions after atrial septal occlusion.

**Figure 4:**
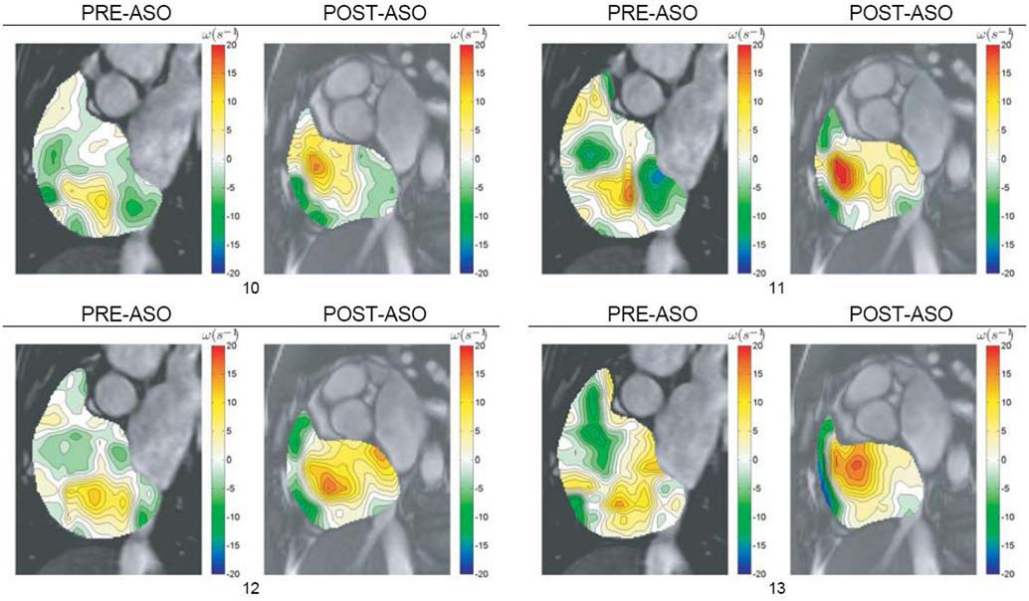
Vorticity visualization of right atrial flow pre- and post-ASO. Vorticity contour maps are superimposed onto cardiac MR images and presented. The vorticity representation by the flow map in color codes can be referenced against scales (in metric units of per second) on the right hand side. The range of the vorticity given by *ω*_Min_ to *ω*_Max_ is stated below each result set with the subscripts 1 and 2 representing pre- and post-ASO conditions respectively.

**Figure 5:**
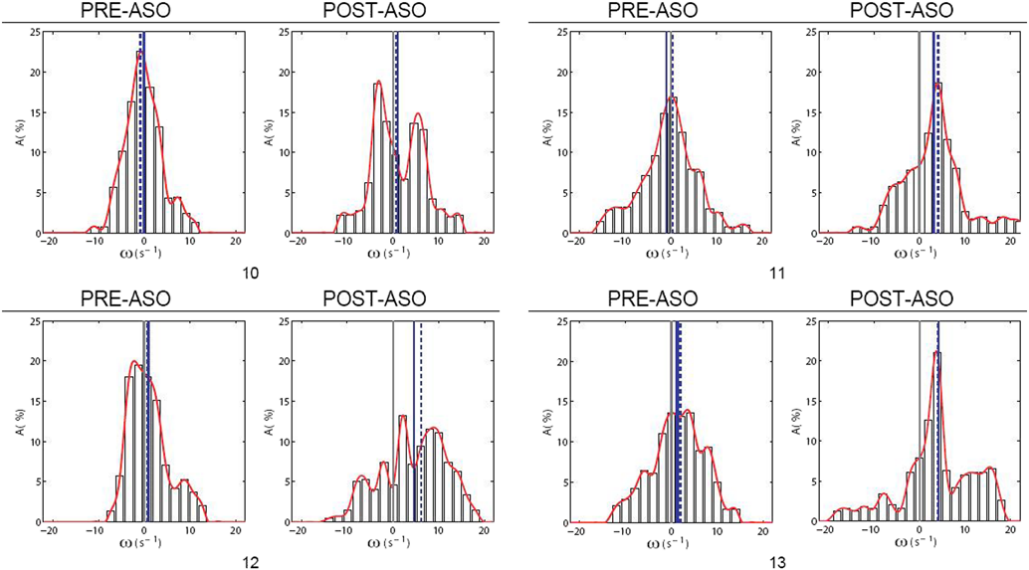
Histograms of vorticity maps for right atrial flow pre- and post-ASO. The histogram represents the distribution of vorticity values in a map. Statistical parameters used include means *ω*_1_, *ω*_2_ and standard deviations *σ*_1_, *σ*_2_ obtained from the histograms of the vorticity maps, with the subscripts 1 and 2 representing pre- and post-ASO conditions respectively. The mean is a gauge of the average angular velocity throughout the flow, and the standard deviation measures the variability of vorticity measures.

We show the mapping of vorticity component normal to the measurement planes, i.e. in the principal direction of the most significant planar flow rotation (Figs. 4 and 5). From these results, we observe that the septal occlusion produces two significant effects. The first one is that it leads to an increase in strength of a single dominant counter-clockwise vortex within the cardiac chamber. Secondly, it reduces the concentration of clockwise vortical regions at the same time. For the post-occlusion condition, a strong negative vorticity region exists adjacent to the chamber wall that has less curvature relative to the septum that is associated with flow of positive vorticity. This is due to the quick motion of blood parallel to the endocardium of a relatively motionless and flat chamber wall. Consistent with this effect, there is a broader distribution of absolute vorticity in the right atrium for the same patient and at the same cardiac phase after septal occlusion. This deduction can be supported using statistical properties of the vorticity distributions. We note the atrial enlargement pre-occlusion, which may in part explain the reduction on the strength of the vorticity concentrations before occlusion. Another possible cause of the differences between the pre-and post-occlusion cases is the presence of left-to-right shunting of blood that disrupts the normal dominant vortex to introduce vortices of a smaller scale (Movie S1).

The statistical properties of vorticity maps for flow maps for pre- and post-ASD pertaining to cardiac phases based on frames of 10 to 13 (out of 25 time frames) are presented in histograms for comparison. The averaging of these properties is performed and illustrated using histograms as well.

**Figure 6:**
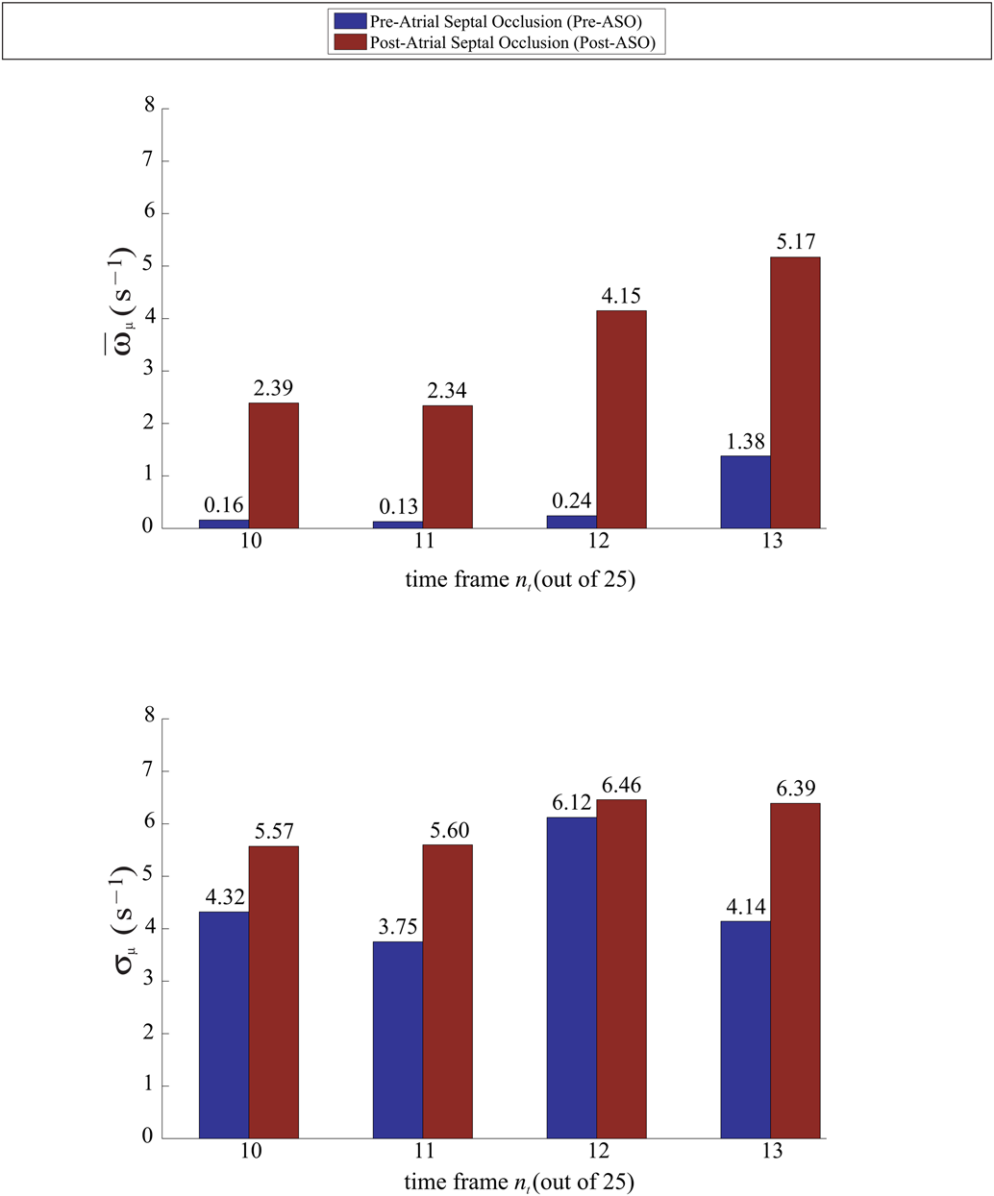
Time-variation of vorticity properties for pre- and post-ASO flow maps. The results show that there is a difference in pre- and post-ASD vorticity maps statistically. Parameters such as mean *ω̅* as well as standard deviations *σ* are based on histograms of the vorticity maps pertaining to time frames *n_t_* = 10 to 13 out of 25 frames in a cardiac cycle. Both averaging of means <*ω̅*> and of standard deviations <*σ*> are shown to be higher for post-ASO vorticity flow maps.

For case study of one patient with ASD, the vorticity mean and variance of the right atrial flow map for post-ASD closure are consistently higher for the selected phases than those for pre-ASD closure (Fig. 6). These statistical parameters can be used reliably to gauge the relative strength of vortices in the atrium pertaining to the pre- and post-ASO conditions. The results in this study show that we are able to use flow imaging and the statistics of vorticity maps at selected time frames of a cardiac cycle to find a difference in flow patterns before and after surgical intervention.

### Summary of Investigation

**Figure 7:**
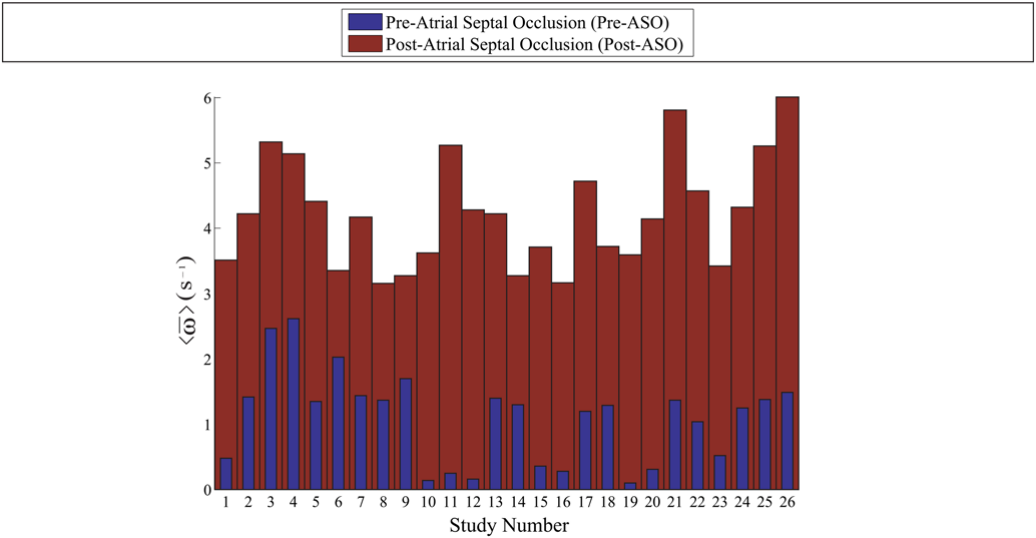
Summary of ASD investigation. The histograms depicting the vorticity comparison for pre- versus post-atrial septal occlusion show that there is a distinct difference between the temporal average of the vorticity means based on a set of selected time frames. Twenty-six patients are used in the framework presented in this study. This investigation may be able to show that the application of vorticity statistics is useful in characterizing abnormal flows in a heart chamber with septal defect.

We carried out the same experiments on the twenty-six patients. Temporal averaging of the ensemble vorticity means based on four or more selected time frames (when the vortices are the strongest) is performed. The results are summarized in Figure 7.

The higher strength of the vortices in the right atrium after an effective septal occlusion can demonstrate that the original vortical flow has indeed been disturbed in the event of left-to-right shunting of blood into the right atrial blood pool.

## Discussion

At this stage, the developed system in our study meets a currently unmet requirement of the medical imaging service industry by providing a non-invasive and fast method for analyzing blood flow that can be used for a number of purposes such as an investigative or prognostic tool for patients at risk of abnormalities of the heart. It can also measure blood flow through artificial cardiac devices *in vivo* at a magnetic strength of not more than 3 Tesla [22].

The exploitation of intensity-based mapping from magnetic resonance imaging data to perform cardiac flow tracking is novel, as there is currently no way to computationally predict flow velocities from these images without encoding of velocity information. The technique lies in the use of commonly used equipment, the MRI machine based on the SSFP MR imaging technique [21], to generate a series of images in spatial as well as temporal dimensions, and post-processing them to measure MR fluid flow in real time. Certainly, individual components of the system (e.g. use of the optical flow algorithm on magnetic resonance images) are not new, but the unique combination of these techniques, and the adaptation of multi-resolution motion tracking used in our study is the key novelty.

Other alternative solutions lie in the use of phase contrast MR imaging, coupled with well-established flow field visualization methods to analyze fluid flow. However, velocity-encoding based on phase shifts in nuclear signals usually take longer scan duration than typical MR imaging. As there are few investigations to computationally measure blood flow using SSFP MRI, the system arising from our study will provide a solution to a currently unmet need. Such a strategy can transfer most of the processing time, by reducing the scan sequence, to the flow prediction and visualization post-processing stages. In addition to this approach, we are able to characterize vortices in heart chambers and establish a relationship between atrial vorticity and a septal defect. As such, there is clear potential of the developed system in its utility as a prognostic and/or investigative tool for assessment of cardiac abnormalities parallel to examining MR images. Of course, this framework can be translated for studying other cardiac abnormality as well but it is beyond the scope of this work to consider further future applications.

Measurement of vorticity based on the MR fluid motion field is performed. By characterizing flow using these parameters, and statistically presenting them for every phase of the flow, we are able to extract more information from the flow model [32]. Flow quantification needs to be carried out so that it can have research value and clinical usage. However, no detailed and quantifiable flow analysis has ever been documented from cardiac blood flow field for diagnostic usage. Even though it may be interpretive to analyze the flow quantitatively using planar measurements, it will be inaccurate because the actual flow is three dimensional. However, we have demonstrated that examination and measurement in two dimensional planes is sufficient in characterizing cardiac abnormalities. Transthoracic and transesophoegal echocardiography are now regarded as the standard for ASD assessment, but a reliable set of cclinical trials can be the focus of a future study to demonstrate substantial value in using cardiac MRI flow analysis.

MRI measurements of blood flow in a heart chamber have been described as an illustration. The developed framework may be extended to assess flow in the other heart chambers or cardiovascular structures. It can conveniently predict flow information with superior processing speed to improve our understanding of blood motion within the heart, which may have implications in the study of blood circulation efficiency. This is extremely useful when MRI scanning is limited by the absence of a velocity-encoding protocol. The implemented study is neither to show with high accuracy the existence of vortices or flow structures in the cardiac chambers nor to verify what has already been established in earlier studies. Rather, it aims to highlight feasibility of the framework for visualizing the flow as well as measuring or comparing vorticities, and with high system versatility to be executed on future batch of case subjects.

Extension of magnetic resonance imaging to the study of flow phenomena is not new, and can be applied to problems of direct relevance to multi-phase flow analysis. The state-of-the-art in magnetic resonance flow imaging studies is discussed previously [23], [24] and the framework can be applied to investigate cardiac blood motion as well. Emerging technologies such as magnetic resonance global coherent free precession [25] can provide a new platform for the usage of MR fluid motion tracking, which generates a potential scheme for cardiac flow tracking now and in the future. Such MRI protocols can be applied to blood spin labeling techniques for acquisition of multiple temporal slices of gray-scale intensity blood flow or cine-angiograms without a contrast agent and use of ionizing radiation [26]. The movement of blood can be tracked as a result of processing such information using fluid motion estimation. Sample global coherent free precession images provide information about vascular function and morphology just as SSFP MR images do. Moreover, it provides a more definite contrast of spin labeled dynamic blood into the non-spin labeled region because of the tagging of blood protons every few milliseconds as they travel through an arbitrary region in space.

Some scan sets can be ineffective for fluid motion tracking. There are a variety of reasons for the inability to develop flow fields from them, such as low image resolution, presence of ghosting artifacts due to respiration during scanning, and absence of vortices that are large enough to be quantified.

It is difficult to achieve high accuracy velocity mapping with low scan duration at the same time. The method that is proposed trades imaging speed with accurate flow field scanning, but still achieves a reliable flow analysis for the cardiac abnormalities. This ultimately leads to flow evaluation for septal defect patients using the available SSFP magnetic resonance images without performing the additional and time-consuming velocity-encoding MRI.

## Methods

This section is based on the description of the engineering methodology and investigation procedures that are carried out. Clinical scans were performed on twenty-six patients diagnosed with atrial septal defect for pre- and post-occlusion conditions. However, to maintain our focus of the importance of biomedical engineering development in this work, only one patient was selected for demonstration of the technical concepts.

### Ethics Statement

Human studies were approved by the Royal Adelaide Hospital Committee and by the Institutional Review Board. MR imaging was conducted in accordance with guidelines defined by the Wakefield Hospital to achieve safe and reliable scanning. The experiment was approved specifically by the ethics committee. Written consent was obtained from each case subject after the imaging procedures had been conveyed.

### Magnetic Resonance Fluid Motion Tracking

There are important and challenging issues in developing a system for diagnosis of cardiac defects based on flow information that is measured. The multidisciplinary approach adopted here highlights a unique combination of machine vision and concepts from particle image velocimetry, image segmentation, and computer visualization. Since the optical flow algorithms are implemented in our approach, we termed this motion-based flow estimation method as a fluid motion estimation scheme; and because MRI is used, we shall classify the technique as a class of magnetic resonance image velocimetry (MRIV).

The application of machine vision algorithms to post-process medical images offers the potential for velocity measurement to become a commonly accessible and technically viable technology. The determination of flow velocities may be based on the intensity shifts of the pixels using the optical flow algorithm. The assessment of flow is accurate provided that a good time resolution is present. However, the number of phases per cardiac cycle will be dependent on the capabilities and specifications of the particular medical imaging device used.

Our proposed technique is specific for characterizing blood flow movement in cardiac structures. The global estimation of flow velocity vector field over the whole image provides useful information on non-laminar flow quantification within the pathologies. The cardio dynamics information that is based on flow analysis and visualization of blood offers potential for the detection and quantification of heart valve failure, septal defects as well as myocardial malfunctioning.

#### Asynchronous precession of proton in magnetic resonating blood

We now examine the theory governing the concept of void signal registration due to turbulence within fluid where atomic nuclei have been aligned either parallel or anti-parallel to a powerful and uniform magnetic field. As high energy nuclei relax and realign, they emit energy with certain properties that are recorded to provide information about the medium. Image contrast is created by weighting the energy signal during realignment of the nuclear spins with the magnetic field. A signal image is then generated as a result of this quantum mechanical activity.

**Figure 8:**
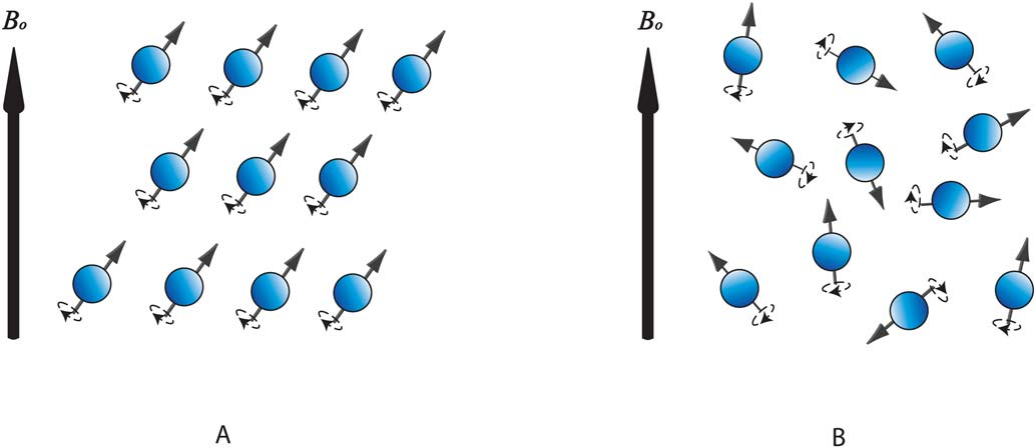
Nature of precessing blood proton spins. Hydrogen-based molecules in blood maintain coherent spin phases in static fluid (as shown in subfigure A), but are distributed chaotically in turbulent fluid and this disrupts the alignment of proton spins from their order (as illustrated by subfigure B). Due to the incoherent or random precession of spins, the net magnetization is weak and gives a low MR signal intensity. Therefore regions of blood at different levels of turbulence appear with contrasting pixel intensities in the cardiac MR image. The groups of incoherently precessing proton spins move along with the global blood flow within the cardiac chamber, and presence of intensity flow, which coincide with the blood flow, can be observed from time-dependent sequences of MR images.

As the fluid is transported, the same signal from nuclei that is retained within the fluid follows the displacement. In a turbulent flow, the diffusion of magnetic moments occurs [27]. Protons in the hydrogen nuclei of molecules can be given a phase spin so the de-phasing (asynchronous precession) of spin due to turbulence in flow (Fig. 8) gives a low MR signal during imaging [28]. Low MR signals are represented as higher pixel intensities in the MR image (Movie S2). The proton spins de-phase in the transverse plane when the RF pulse is terminated. Other than the cause of uncontrolled diffusion of magnetic moments in turbulent fluid, this de-phasing can also be due to intrinsic non-homogeneities of the magnetic field as a result of magnetic susceptibility changes at tissue interfaces.

Therefore, there is a reduction in the signal registration on the image. Effective diffusivity of spin protons at this point is represented by a reduction in signal intensity in the image [27]. Because the diffusivity follows the movement of the fluid in a channel, there exists an intensity of change in the direction of flow. The intensity contrast of the diffusion in the image thus becomes greater as the higher speed of the fluid flow increases the disorder in the fluid.

It is also important to emphasize that in the heart chambers, the nuclear spins may move perpendicularly in and out of the imaging plane. Therefore, spins that receive the original excitation may, in turbulent flow, not experience magnetic resonance gradient refocusing. Likewise, spins that do not receive the original excitation may in fact move into the imaging plane after the RF excitation pulse, and since they were not quantum mechanically stimulated to begin with, no MR-signal may be returned. Extrapolating this concept further, some signal loss due to fast flowing jets are likely due to spins moving too quickly to be excited and refocused, creating signal voids.

Due to the inhomogeneous presence of asynchronous proton spins, nuclear signals emitted from dynamic fluid displays on MR images as variation patterns of intensity depicting the blood flow movement. It may be worthwhile mentioning that poor temporal and spatial resolution imaging may blur the observation of blood movement between consecutive images. At some phases of scans, the presence of low-turbulent regions may also weaken the intensity contrast variation in blood images, so that visual tracking of blood motion declines in accuracy.

#### Tracking of MR signals

The optical flow technique [29] extended to perform multi-resolution motion prediction [30], [31] from MR signals is useful for evaluating blood flow properties in heart chambers. Therefore, optical flow evaluation of MR images is able to provide flow field measurement. The global estimation of flow velocity vector fields across the whole image provides useful information on vorticity which defines the vortices within regions of a relevant pathology. This type of approach has been used in imaging and computer vision previously for motion analysis of rigid structures, but we modify and apply it to compute fluid motion based on temporal signals from MR images. The MRI based fluid motion estimation framework is termed as MR fluid motion tracking [32].

The implementation of the multi-resolution Lucas Kanade optical flow technique [30], [31] on the MR image grid, based on sectional scan of a heart, allows non-laminar flow velocity field, on a two dimensional velocity grid, to be measured. The global estimation of flow velocity vector fields within the region of interest provides useful information on non-laminar flow quantification within the heart chamber. Such estimation of blood behavior can provide information on myocardial ischemia and infarction. The technique can also be introduced for evaluating blood flow from MR images of heart chambers or arterial flow channels. The study of vorticity, shear and normal strain distributions, computed from the velocity field, can be used to provide insights into the behavior of the flow. The global estimation of vorticity fields also provides useful information such as formation and evolution of vortices within heart chambers. A useful feature of our developed system is the computation and presentation of these properties temporally in two dimensions. These can also be presented for multiple slices of scan simultaneously.

It may be worthwhile mentioning the parameters that will affect tracking performance. Presence of asynchronous spins within a scan image contributes to track features within a region of interest. Therefore, significant degrees of spin coherence possessed by a group of protons, low resolution and poor signal-to-noise ratio of imaging will reduce the number of track features and causes inaccurate tracking [32].

#### Multi-resolution Motion Estimation Algorithm

In a multi-resolution, optical flow scheme, the pyramidal optical flow method incorporating a multi-scale approach has been applied to support multi-scale fluid motion and to improve accuracy and robustness [31]. A top-down estimation of the flow using an image pyramid is performed, with the apex representing the MR image at a coarse scale. The calculation of the optical flow field at every image level is based on the optical flow constraint [29].

**Figure 9:**
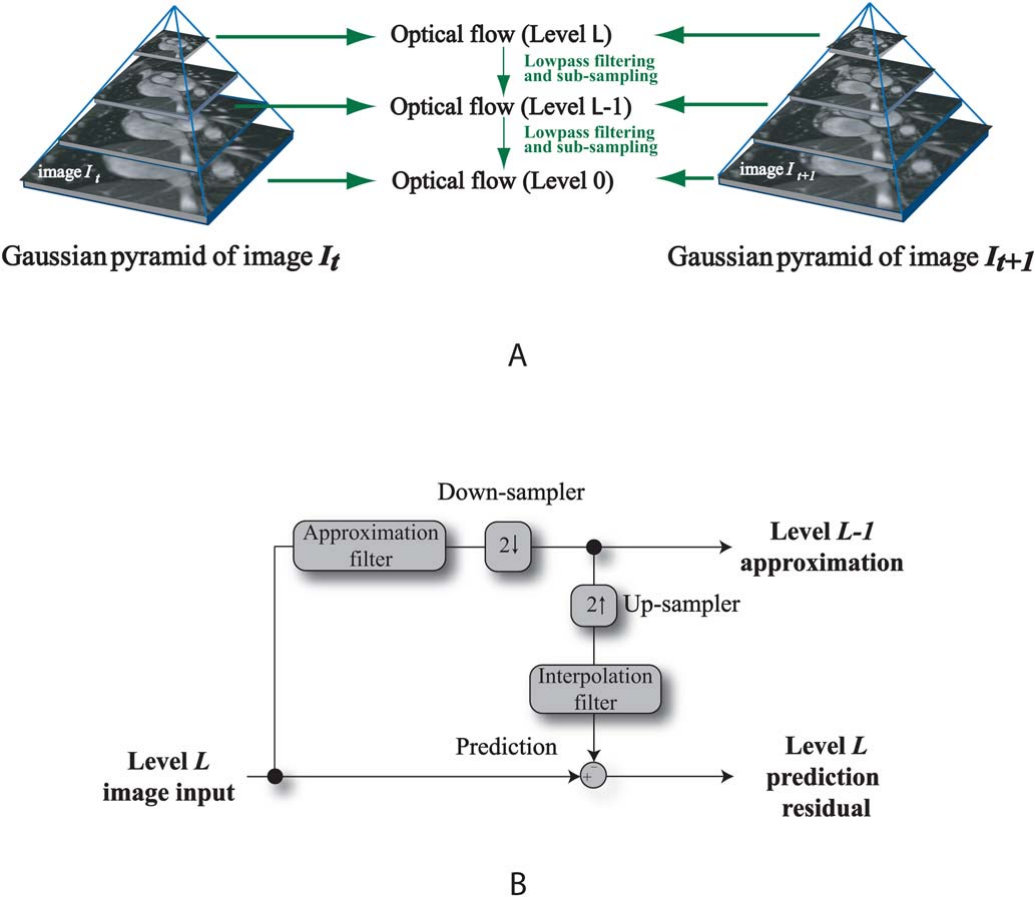
Multi-resolution motion estimation using pyramid implementation. Diagrammatic view of the Gaussian pyramid with optical flow applied onto every image level (0 to *L*) is presented in subfigure A. Each level in the pyramid is a sub-sampled version of the level below. In the first step, the optical flow between the top level images is computed. We project the computed coarse-level flow field onto the next finer pyramid level and continue this at each level of the pyramid until the finest pyramid level has been reached. The system block diagram in subfigure B gives an illustration of the algorithmic operation of this pyramidal implementation.

Computational results from this level are passed to the next level and this is continued and based on the estimated flow at the preceding scale until the original scale is reached. We refer to the diagram in Figure 9 to illustrate the computational aspect of pyramidal optical flow. The accuracy of motion estimation critically depends on the magnitude of image motion. In fact, depending on the spatial image frequency, very large motions even may cause aliasing. For a fixed global velocity, spatial frequencies moving more than half of their period per frame may cause temporal aliasing [33].

Aliasing can lead to significant errors in velocity and vortex strength assessment, which affects the accuracy of the method in determining vortex strength. We sought to devise a way of removing outliers in the flow vector field. Optical flow vectors that pertain to very large pixel displacements relative to that of the pixels in its adjacent region are isolated using the median test [34]. We perform clustering of optical flow vectors with magnitudes above a user defined threshold. The flow vectors within a cluster are classified as outliers provided that two conditions are fulfilled: (1) the difference between the vector magnitude and the median magnitude in the flow field exceeds a threshold that can be arbitrarily set; and (2) the number of vector items within the group that is encapsulated by a sampling window size that met the first condition do not fall below a specific value.

Voids due to the removal of these outliers must be filled in with new vectors for the flow to be continuous. We devised a simple approach of growing vectors using a flow field synthesizing approach. This technique is analogous to the occlusion fill-in algorithm used in texture synthesis by non-parametric sampling except that the sampled elements are flow vectors instead of texels (texture elements). We can also apply reduction in resolution to the flow field. The averaging of vectors within interrogation windows results in a lower resolution of flow field but also can help in smoothing of flow data by averaging the high magnitude vectors of these outliers and smaller vectors in its neighborhood regions.

### Visualization of Blood Vortices within the Heart

Vorticity *ω* (in units of ms^−1^) is defined as the curl of velocity *v* whereby *ω* = ∇×*v* and is a representation of the orientation and angular velocity of local rotation [34], [35]. The display of a two dimensional vorticity map of a single vortex based on its velocity field can be used to indicate the vortex core and its rotation. By analyzing the vorticity field of a vortex, the strength and length scale of the vortex can be characterized.

A definition of vorticity is provided by examining the magnitude of rotation of fluid about a specific examined point [34]. The measurement of a three dimensional vortex structure is dependent on the angle of the measurement plane. When vortices are skewed with respect to the measurement plane, the results are affected by the skew angle [14]. However, presentation of resolved vector components of spiralling flows or skewed vortical flow in a three dimensional space is visually complicated and will make the analysis of vorticity difficult. We see in this study that visual examination of vortices in cardiac MR images of the human chamber using two dimensional slices of the scan is sufficient. We compare magnitude and occupancy of a single vortex on a specific slice and phase of the cardiac cycle for a patient who has been scanned pre- and post-septal occlusion.

This section describes the flow characteristics of the vortices within the right atrium pre- and post-atrial septal occlusion (ASO). We base our analysis on quantitative data pertaining to vorticity calculations and support the flow scenario with superimposed qualitative data such as velocity vector plots and streamlines. The purpose of our analysis is to reach a conclusion that a difference in the vorticity after surgical intervention is visible, and we use our implemented technique to support this claim. The characteristics for the vorticity map can be presented in histograms as an indication of vorticity magnitudes represented by color pixels in the flow grid. Swirl within segmented right atrium for pre- and post-ASO at three scan slices of heart are presented (Fig. 2,3,4 and 5) and comparison of vorticity for both conditions is carried out and shown (Fig. 6).

In this study, we focus on two sets of investigations, one on the right atrium of an atrial septal defect (ASD) subject before treatment, and the other set on that of the same patient after surgical atrial septal occlusion (ASO). The flow information from these two situations can be used to explain the behavior of vortices that develop in the right atrium of a heart with a septal defect before and after ASO.

### Investigation Procedures

#### Sample Case Study Subject

The case subject is a male patient, aged 18 years at the time of this study. The subject had an ASD and was assessed using cardiac MR imaging. Implantation of the septal occluder device in the patient was performed 79 days later and another scan was carried out 210 days after the ASD occlusion. Cardiac MR imaging is performed on the patient using a Siemens Sonata, 1.5 Tesla, model–syngo MR 2004A scanner with Numaris–4, Series No: 21609 software. Steady-state free precession cine-MR imaging was performed using contiguous slices in short axis views through the ASD. All images were acquired with retrospective gating and 25 phases (from time frames *n_t_* = 1 to 25) for each slice are obtained.

SSFP magnetic resonance imaging is used to scan the patient before and after atrial septal occlusion. Acquisition parameters include: TR = 47.1 ms, TE = 1.6 ms, FOV = 298×340 mm at matrix of 134×256 pixels for pre-septal occlusion scans and TR = 40.3 ms, TE = 1.2 ms, FOV = 260×320 mm at matrix of 156×192 pixels for post-atrial septal occlusion scans.

#### Engineering Methodology for Flow Analysis

Cardiac MR image slices of the patient that correspond to diastolic phases of the heart are selected. We observe the vortices that are generated pre- and post-septal occlusion at the selected phases. The right atrium was enlarged due to a left to right shunt, and the changes in the signal intensity of blood using MRI are more easily detected than in the other chambers. Note that we have chosen the right atrial flow for analysis, since this is the chamber into which we can observe the effect of blood shunting from the left atrium. A region of interest with width of 120 pixels and height of 150 pixels is truncated from the selected slice showing the sectioning of the right and left atria, where the septal defect is most apparent. This is to be displayed for qualitative examination.

Statistical histograms are produced by counting the number of frames with the specified range of vorticity values and then consolidating them to create a frequency versus vorticity value plot. The frequency of the image frames can be normalized to become the area *A*(%), which gives the percentage of the entire flow region.

**Figure 10:**
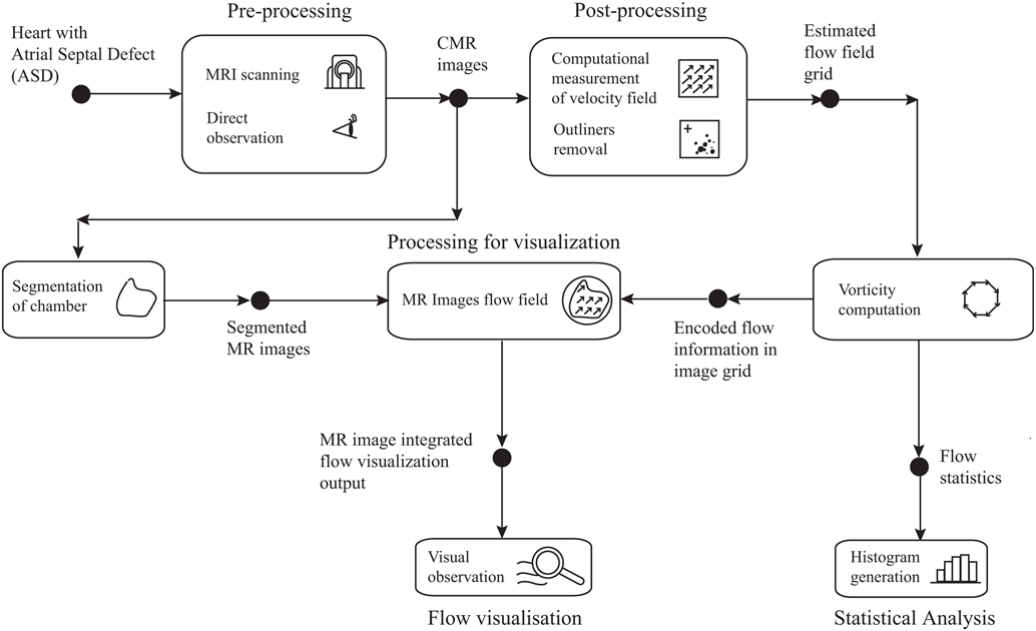
Cardiac vorticity visualization system for ASD investigation. This system is specially constructed to review vorticity characteristics in patients with atrial septal defect (ASD) and relies on computational post-processing of MR images. The vorticity visualization system flow shows data processed by blocks depicting the MR imaging which provides data for generation of flow field flows. Flow visualization is achieved by field display using vector plot with the superposition of MR intensity images. Statistical analysis is supported by histograms of the vorticity maps.

We implement a vorticity visualization system dedicated to the examination of flow behavior in the heart chambers. We examine the system flow stages from imaging of patient with atrial septal defect to the visualization of cardiac flow field and statistical analysis of the vortical flow in atrium of the imaged heart (Fig. 10). Flow visualization is performed at the post-processing stage whereby vector plot is computed based on image intensity motion vectors for every pixel. The segmentation is required for isolating the region within the chamber for observation (Movie S3) and also for selecting only the relevant vorticity values in the atrium to be used statistically for analysis.

### Validation of technique

It is necessary to establish a form of measure for reliability of a system to accurately develop vorticity maps of flow field imaged by MR fluid motion tracking technique. The reliability of a flow field prediction can be defined as ratio of the true variance to total measured variance of vorticity maps [36]. In this respect, based on an arbitrary time frame of the cardiac cycle, a vorticity map from the flow field by phase contrast MRI velocimetry is assumed as the true data. The map produced from flow field developed by MR fluid motion tracking (hereby termed the predicted map) has inherent noise and is a combination of the true and error data.

#### Vorticity Field Differencing System

**Figure 11:**
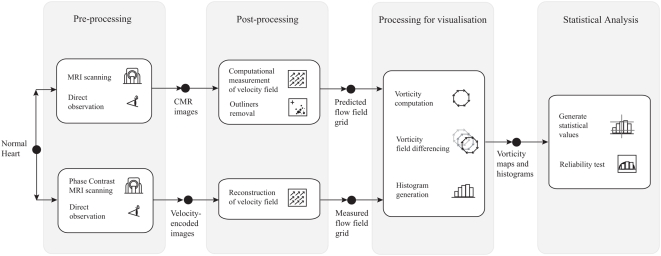
Reliability test system for MR fluid motion tracking based on vorticity differencing. This system is specially constructed to calibrate the performance of MR fluid motion tracking against the well-established phase contrast MR image velocimetry system. Pixel by pixel subtraction followed by scaling is performed to produce the vorticity field difference (or error) map.

Using Figure 11, we demonstrate the systematic implementation of vorticity field differencing based on four stages. The first stage starts with scanning of a normal heart with steady-state free precession MR imaging and on a separate occasion, the same scanning using phase contrast MR imaging. During this pre-processing stage, blood motion information is encoded within images in a different manner. The cardiac MR images contain temporal positions of magnetic resonating blood, whereas the phase contrast images have local blood velocities encoded within them. In the visualization processing stage, both field grids are further processed to give their respective vorticity field, which are then differenced pixel by pixel.

#### Phase Contrast MRI Scan Procedure

For this study, velocity-encoded MR imaging was performed using a Siemens Sonata, 1.5 Tesla, model—syngo MR 2004A scanner with Numaris—4, Series No: 21609 software. Cine-MR imaging was conducted using one slice in short axis views through the atria. All images were acquired with retrospective gating and 25 phases.

#### Parameters for Data Analysis

The histogram of a vorticity map with vorticity values in the range [0, *L*−1] is a discrete function *h*(*r_k_*) = *n_k_*, where *r_k_* is the *k* ^th^ vorticity value and *n_k_* is the number of pixels in the flow map having vorticity value *r_k_*. Statistical quantification of the blood vorticity map in the right atrium is performed by translating all the scalar values into histogram format. The size of right atrium is different for every phase. Normalization is performed by standardizing the total count of pixels within a segmented region of interest to create an equal integral area under the frequency plot of the flow map. The total counts correspond to the number of pixels representing the atrium per slice.

The velocity fields of the cardiac flows are calibrated by using the MRI scan properties as indicated in Table 1. From Table 2, the width of flow image is 120 pixels, and we have set the velocity interrogation window to a 3 by 3 pixels frame. We observe vorticity flow maps pertaining to time frames *n_t_* = [17, 18, 19, 20] out of a maximum of 25 frames in a cardiac cycle for a vorticity sampling window size of (19×19) pixels.

**Table 1:** MR imaging properties.

Symbol	Quantity	SSFP MRI Scan	Phase Contrast MRI Scan	Units
*p*	Pixel spacing	1.67	1.54	mm/pixel
*t_s_*	Trigger time interval	35.72	29.43	ms
*s*	Slice thickness	6	6	mm

The properties of SSFP MRI and the phase contrast MRI scans are used to calibrate flow maps, and present the velocity fields in metric units.

**Table 2:** Vorticity measurement properties.

Symbol	Quantity	Value	Units
*X*	Image width	120	pixel
*Y*	Image height	150	pixel
*W_v_*	Resolution of velocity grid	3×3	pixel

The vorticity flow maps can be determined from the velocity fields of cardiac flows. Properties of these velocity field image parameters are used to calibrate the vorticity maps as well as indicating the sampling vorticity mask size in metric units.

#### Calibration of Vorticity Sampling Window

In this study that uses a normal atrium, we assume the true-score variance *σ*_TRUE_^2^ to be the equivalent of the vorticity variance based on a true map measured by phase contrast MRI. We note that the error variance with the expression *σ*_ERROR_^2^ is the variance of an error map, which is produced by differencing the predicted and true vorticity maps. The components of this error can come from existence of other smaller vortices along with the dominant single vortex in the right atrium that escape detection by the fluid motion estimation due to inferior quality of tracking features. The reliability of flow field prediction using MR fluid motion tracking defined as *ρ* is the ratio of true vorticity variance *σ*_TRUE_^2^ to total measured variance given by *σ*_TRUE_^2^+*σ*_ERROR_^2^. Ideally, if the system can generate flow fields that correspond close enough to those that are measured by a gold standard imaging modality such as phase contrast MRI, the error tends to approximate zero and reliability becomes one.

We vary the vorticity sampling window parameter from (3×3) to (33×33) pixels with increments of (2×2) pixels at each interval to chart the reliability of flow measurement using MR fluid motion tracking versus phase contrast MR imaging technique. We observe vorticity flow maps pertaining to time frames *t* = [17,18,19,20] for a series of sampling window sizes in Figure 11.

**Figure 12:**
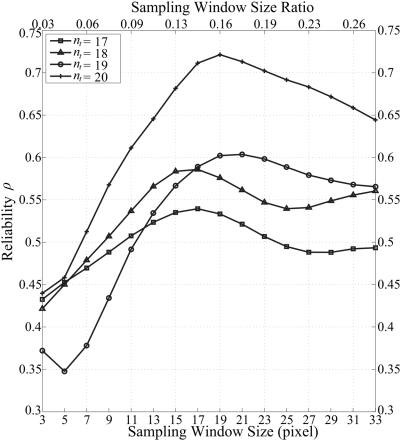
Reliability study using predicted and true variance. Reliability for the predicted MR fluid motion field data, *ρ* with respect to the phase contrast MRI data (taken as the true data) is computed. The variation of vorticity sampling window size can affect the reliability of computational measurement of the vorticity.

Implementing an appropriate sampling window size for vorticity measurement can improve the visualization of the vorticity maps to be as close as possible to the map that is based on phase contrast MRI flow. Generating the variation of its reliability with respect to the sampling window size can give us an indication of the ideal sampling window size value. Based on the results (Fig. 12), we choose a sampling window size of (19×19) pixels for our vorticity field generation.

#### Comparison of Imaging Modalities

**Figure 13:**
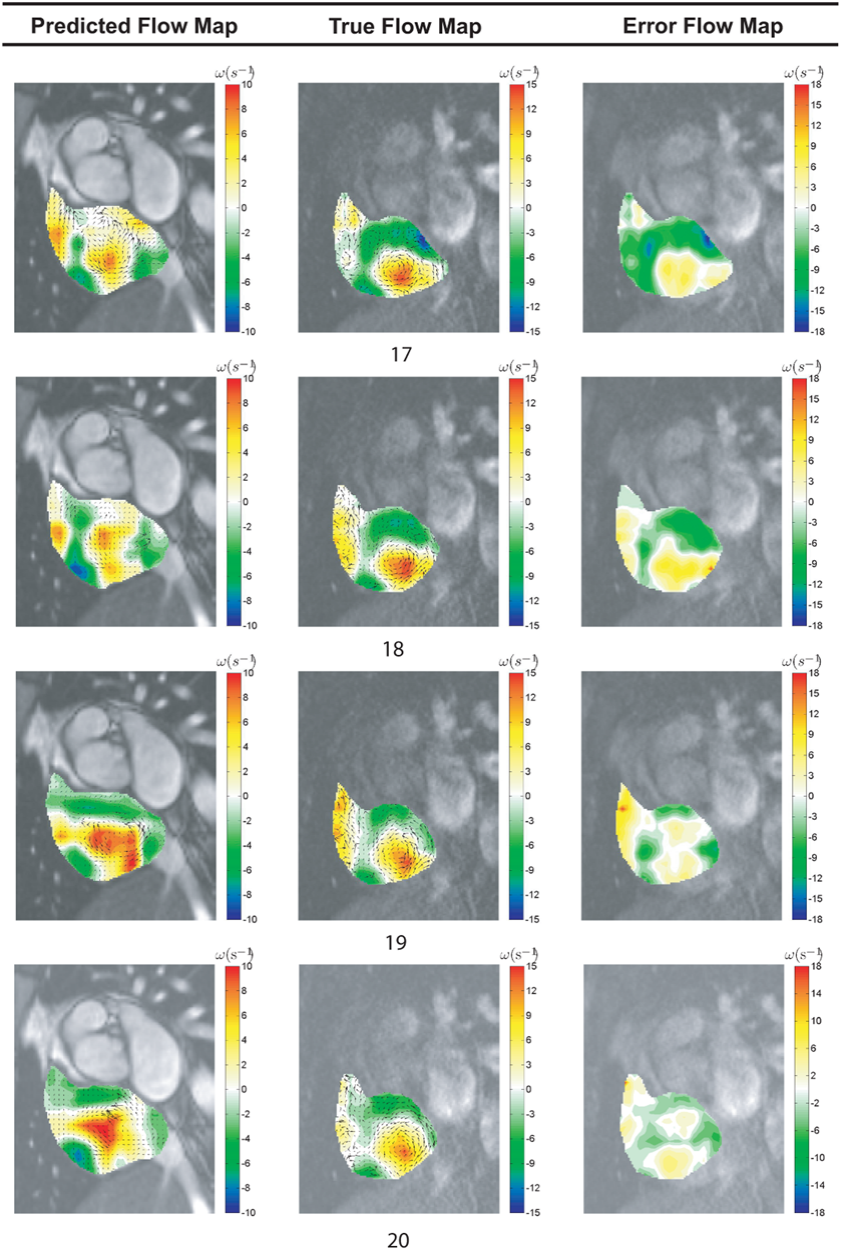
Vorticity differencing based on MR fluid motion and phase contrast magnetic resonance image fields. Predicted flow fields generated by MR fluid motion tracking are verified against the measured ones based on phase contrast magnetic resonance image velocimetry. The former technique post-processes temporal MR signals to compute flow, while the latter velocimetry system processes the signal during scan and encodes the velocity information in images. The set of results presented here illustrates the difference between blood flow fields in the human right atrium, of which one is predicted and the other is accurately measured. Therefore, their vorticity map differences can be taken as the deviation of MR fluid motion field from the true flow field. We demonstrate flow field differencing using time frames, *n_t_* = [17, 18, 19, 20] out of 25 frames in a cardiac cycle.

Motion prediction based on MRI generates flow field of the blood pool that can have limitations in terms of accuracy if the signals emitted by the blood vary poorly in contrast spatially. Therefore, the motion estimation algorithm needs to be robust and reliable enough to enable good prediction of the blood motion globally. The velocity-flow data also needs to be processed a second time to provide differential flow measures such as vorticity. It may be worthwhile to highlight that phase contrast MRI can produce much more accurate flow information, however; scanning time is significantly higher than standard MRI. Although the tracking system trades off accuracy with processing time, it can deliver a good prediction of flow structures within the heart (Fig. 13).

The measurement of a three dimensional vortex structure is dependent on the angle of the measurement plane. When vortices are skewed with respect to the measurement plane, the results are affected by the skew angle. However, presentation of resolved vector components of spiraling flows or skewed vortical flow in a three dimensional space is visually complicated, and this causes the analysis of vorticity to be difficult.

Considering that the SSFP and phase contrast magnetic resonance imaging are performed on two separate occasions with slight dissimilarities in the planar imaging configuration, perfect alignment of the right atria regions is difficult. Therefore a near zero error map of the vorticity maps resulting from the flow images of MR fluid motion tracking and velocity-encoded MRI is hard to achieve. Based on this discrepancy, we have to take into account of additional error contributed by the imperfect comparison scheme. These issues explain the large error margin exhibited by MR fluid motion tracking. But the qualitative observation of the velocity flow field as well as vorticity differencing results may still provide useful information for cardiac flow assessment.

We also note the low resolution of images (120×150) pixels or (184.8×231.0) mm^2^ that are used here in this experiment. If higher signal-to-noise ratio imaging with superior resolution is implemented, the tracking of the magnetic resonating fluid will generate flow fields that are more accurate. In any case, the aim is to identify the location and strength of vortices can be achieved by MR fluid motion tracking. Although the flow may differ strictly in terms of vector comparison that is applied throughout the vector plot area, the general fluid paths are qualitatively observed to be similar, and the large-scale vortices can still be analyzed using an appropriate vorticity mapping.

## Supporting Information

Movie S1 Use of SSFP magnetic resonance imaging to detect atrial septal defect (ASD). Note the multiple vortices in the right atrial flow within the heart before atrial septal occlusion (ASO).
(0.33 MB MOV)

Movie S2 This is the SSFP magnetic resonance imaging of the atrium after atrial septal occlusion (ASO). We observe a faster blood rotation based on the intensity contrast exhibited by asynchronous blood proton spins for a series of time frames. There is a dominant vortex in the right atrium.
(0.34 MB MOV)

Movie S3 Segmentation using semi-automatic active contouring based on the Kass Snake algorithm is carried out. Region of interest is defined after atrial contouring. The flow analysis is based on blood within the isolated chamber.
(0.16 MB MOV)
